# Suicidal Ideation and Behavior Among Congolese Refugees in Rwanda: Contributing Factors, Consequences, and Support Mechanisms in the Context of Culture

**DOI:** 10.3389/fpsyt.2020.00299

**Published:** 2020-04-24

**Authors:** Chantal Marie Ingabire, Annemiek Richters

**Affiliations:** ^1^ Research Department, Community-Based Sociotherapy (CBS), Kigali, Rwanda; ^2^ Amsterdam Institute for Social Science Research, University of Amsterdam, Amsterdam, Netherlands

**Keywords:** suicide, Congolese refugees, Rwanda, family conflict, gender equality, economic deprivation, teen pregnancy, cultural dynamics

## Abstract

Concern in one of the five camps for Congolese refugees in Rwanda about suicide attempts and death in 2017 as well as research data pointing to a relatively high incidence of suicidal ideation in this and a second camp in the same period provided the impetus for this exploratory qualitative study. The study explored factors contributing to suicidal ideation, attempts and death; existing support and referral mechanisms; and recommendations regarding prevention and care strategies. Between July and September 2018, 10 focus group discussions were conducted with refugees and representatives of stakeholders working in the camp, and 21 in-depth interviews with refugees who reported suicidal ideations in a previous quantitative survey, two refugees who attempted suicide, and family members of those who reported suicidal ideas, attempted suicide or committed suicide. Findings suggest that while all refugees have suffered from war and violence in Congo and experienced traumatic events before arriving in Rwanda, the pathway to suicidal ideations was often triggered by the circumstances related to their current situation in the context of refugeehood. Almost all respondents who experienced suicide ideations and/or attempted to commit suicide reported poor mental health, a low sense of connectedness/belonging and a high level of perceived burden, which were greater than their desire to live. Family conflicts were found to be an important starting point leading to suicidal ideations and in some cases to suicide attempts and deaths. For the adult population, family conflicts often resulted from the cultural and legal changes experienced after fleeing their home country, misunderstandings of Rwandan gender equality policies, and disagreements about family income management. For youth, a lack of hope for the future was found among boys and girls, and for some girls, suicidal ideations were triggered by poor interpersonal/family relationships due to unwanted pregnancies. Family, community and faith-based support mechanisms were reported as being available but not always culturally sensitive. Psychosocial support services should be improved and expanded to ensure effective psychosocial recovery. Family conflicts related to a lack of family communication and a misconception of gender equality policies should be tackled with attention to the cultural factors involved.

## Introduction

Suicide, defined as an act of taking one’s own life, is a global phenomenon that occurs among people from all ages. According to recent statistics, approximately 800,000 people die due to suicide every year worldwide. In 2016, 79% of suicides occurred in low- and middle-income countries. In that same year, suicide accounted for 1.4% of all global deaths, making it the 18^th^ leading cause of death [World Health Organization (1)]. The relatively scarce research to date on whether or not asylum seekers and refugees are at a higher risk of suicide than other migrant and non‐refugee populations has yielded contrasting findings ranging from no difference to a much higher risk [cf. (1–7)]. Factors contributing to a vulnerability for mental disorders and relatively high suicide rates among refugee and asylum seeker populations include potentially traumatic war experiences, distressing life events, difficult living conditions, restrictions on movement, isolation, being uprooted, the lack of social networks and traditional support mechanisms, challenges to self‐concept and individual and group identities due to legal constraints, a lack of help‐seeking behavior, and uncertain future prospects [e.g. (3, 8, 9)]. The interplay between distant triggers such as mental disturbances, genetic predisposition, and chronic stressors give fertile ground for proximal triggers, namely acute distress, interpersonal conflict, and financial crises that precipitate self‐harming acts and constitute a crucial factor for suicide behavior (9). The study presented here, adds to the small number of studies on suicide among refugee populations in various parts of the world. It also responds to the call by Mars et al. (10) to have more studies, in particular qualitative ones, on suicide in sub-Saharan Africa, where suicidal behavior is an important but understudied issue. Ours is a suicide case study in a specific refugee situation—Congolese refugees in Rwanda living in two camps.

According to the United Nations High Commissioner for Refugees (UNHCR) (11), Rwanda hosts approximately 145,895 refugees and 76,202 (52.2%) are from the Democratic Republic of Congo (DRC). The Congolese refugees located in five camps across Rwanda include those who fled their country in the 1990s, as well as the more recent arrivals in 2012-2013 due to renewed hostilities in eastern DRC. The impetus to conduct a study on suicide in two of these camps was concern among the refugee community, camp managers, and stakeholders about suicidal behavior after December 2017, despite a range of psychosocial interventions conducted in *Nyarugenge* and *Mukarange* ^1^ refugee camps. The main study objectives addressed the perceived risk factors of suicide (i.e. individual, interpersonal, economic, cultural), existing family, community and institutional support and referral mechanisms, and recommendations to inform appropriate prevention and care strategies to be adopted.

The study was action-oriented and should be classified as applied research. Like much research on suicidal behavior, it was not informed by a theoretical perspective. However, a brief analysis of some study results in the *Discussion* section is guided by the “ideation-to-action” framework developed by Van Orden et al. (12) and Klonsky and May (13). This framework builds on the distinction between suicidal ideation and action, which is “especially important when one considers that most people who develop suicidal ideation never go on to make a suicide attempt” (13). Our study confirms this observation. In the study design, we made a distinction between suicidal ideation (suicidal desire, suicidal thoughts) and suicidal action or behavior, differentiated as non-lethal suicidal action (attempted suicide) and lethal suicidal action (deadly suicide or completed suicide). We later found this approach confirmed to the aforementioned ideation-to-action framework.

## Methods

This qualitative study was informed by a comparative quantitative study conducted in 2017 in the context of the implementation of community-based sociotherapy^2^ in Congolese refugee camps in Rwanda. The study documented the outcomes of participation in the psychosocial support intervention in regard to the participants’ mental health [including (non-)existence of suicidal ideations], perceived social support, life coping mechanisms, and state of economic welfare. A quantitative study was conducted between 2017 and 2018 among 98 (50 in Nyarugenge and 48 in Mukarange) and 74 refugees^3^ (37 respondents in each camp) prior to and after their participation in sociotherapy. Using a systematic sampling, participants were selected from the lists of those attending sociotherapy. Subsequently, a qualitative study on suicidal ideations in both camps was performed between July and September 2018. In total, 10 focus group discussions (FGDs) with 86 people were conducted (44 men and 42 women) across both camps. Two FGDs were held with religious and local leaders (including refugee representatives); two with community members, two with stakeholders working in the camps (Ministry of Emergency Management (MINEMA), UNHCR, World Vision, Plan international, Africa Humanitarian Action (AHA), Humanity and Inclusion (HI), Community-based Sociotherapy/Duhumurizanye Iwacu Rwanda as well as the American Refugee Committee (ARC); two with refugee community mobilizers working for Plan International, HI, World Vision and the sociotherapy program; and two with members of the refugee committee of elders and vulnerable people. In addition, 21 in-depth interviews (IDIs) with seven male and 14 female refugees were conducted. IDIs were conducted among the nine refugees who had reported suicidal ideations in the quantitative pre-intervention survey, two refugees who had attempted suicide, and family members of those who a) had reported suicide ideation (n = 3), b) had attempted suicide (n = 2), and c) had died as a result of suicide (n = 4). None of these interviewees participated in the FGDs. Prior to the individual interviews, respondents read and signed an informed consent form and an ethical approval from the Rwanda National Ethics Committee (No.106/RNEC/2018) was obtained. Interviews were conducted in Kinyarwanda, transcribed and translated into English, followed by a thematic analysis according to the research questions. Written consent forms were also obtained for summarized stories highlighted in the *Discussion* section and names have been modified for ethical purposes. Findings were presented according to the study objectives and following the order of importance regarding causes of suicide as perceived by respondents.

## Results

### Incidence of Suicidal Ideations, Attempts and Suicidal Deaths in 2017 and 2018

Data presented in **Table 1** shows that the majority of those who reported suicidal ideations were women, whereas men actually committed suicide. Suicidal ideations were reported in both camps, while all suicidal attempts and actions happened in Nyarugenge refugee camp.

**Table 1:** Incidence of suicidal ideations, attempts and suicidal deaths in 2017 and 2018.

	Suicidal Ideations quantitative pre-intervention data October 2017 N=9	Attempted Suicide qualitative interviews Dec 2017–June 2018 N=2	Suicidal Deaths data provided by family Dec 2017–June 2018 N=4
**Camps**			
Nyarugenge	4	2	4
Mukarange	5	0	0
**Sex**			
Male	3	1	3
Female	6	1	1
**Age**			
<15	0	0	0
15–25	3	0	1
26–50	2	1	1
>50	4	1	2
**Education**			
No education	4	2	0
Primary	2	0	4
Secondary	3	0	0
**Marital status**			
Single	1	0	1
Married	3	0	2
Separated	2	2	1
Single mother	2	0	0
Widow	1	0	0

### Perceived Causes of Suicidal Ideation and Behavior

#### Everyday Distress Related to Refugeehood

Most respondents appreciated the pre-migration life in Congo before the invasion of armed groups, the war that started in the 1990s, and other forms of violence. A large number of families had owned land for agriculture and cattle grazing activities. The high level of insecurity, family separation, family member murders, and loss of property made many refugees ultimately decide to flee to Rwanda. Although the refugees were provided with support for their basic needs in the Rwandan camps, many found it difficult to make ends meet. They also experienced difficulty coping with the memories of their painful traveling from Congo to Rwanda and the losses they had experienced. Suffering due to their past traumatic experiences coupled with current cultural differences and daily life challenges often resulted in tension and conflict at the family level. According to the family member interviews, conflict was prevalent in the families of someone who had had a desire to commit suicide or had attempted suicide. This suicidal desire and behavior resulted from many factors, including polygamy, adultery, misconception of gender equality, lack of proper and regular family communication as well as family income management such as the monthly allowances provided by the World Food Program (WFP). It was also reported that some refugees with suicidal ideation and behavior had previously had familial experiences of suicide (i.e. grandparents, uncles or other family members) as a way of solving their (family) problems.

*These people changed life, but they didn’t change their mind. Problems similar to the ones people face today existed even in their home country. However, today people are isolated, they don’t see a future, even a future for their children. In Congo, if one of the parents was unable to feed his children, he preferred to commit suicide, which is the same here today*. Church leader, Nyarugenge.

*They* [men] *can’t endure the hard life they are living now. They used to support their family, but now they can’t even get money for salt. In some cases, some of them commit suicide. In these hard-living conditions, conflicts tend to arise in families due to poverty and hunger, which sometimes end with suicide. A man who used to drink milk and used to have a word in a society and in his family, experiences that all these things are no longer there*. Male respondent, FGD with refugee committee of elders and vulnerable People.

While multiple and intertwined factors lead to suicidal ideation and behavior, in the section that follows, the factors that put Congolese refugees in Rwanda at risk for suicidal ideation and behavior are presented individually.

#### Loss of Manhood and (Mis) Understanding of Gender Equality

Many Congolese respondents (mainly men) reported changes in gender roles and responsibilities as a result of relocating to Rwanda. In Congo, families were characterized as dominated by a patriarchal system in which men were perceived to be the heads of and providers for their families (wives and children) and responsible for familial order and protection. Arriving in Rwanda, men were confronted with a legal framework in which women’s rights and empowerment are key and serve as a prerequisite to sustainable development (16). Some respondents indicated that the Rwandan legal framework for gender favored women, who back in Congo could be exposed to toxic masculinity and gender-based violence with no legal sanction (17). Some respondents had misconceptions of principles of gender equality as promoted in Rwanda and reported that women think they are superior to their husbands and adopt a conceited attitude. Male refugees in the IDIs and FGDs perceived Rwandan gender equality (focused on women’s empowerment) as a way to disempower them and/or attack their value and dignity, which caused resistance and conflict between men and women. The situation was even harder to accept for men who perceive themselves and/or are perceived by others as no longer being family breadwinners, guides, providers or protectors as highlighted in the quote below.

*The reason that we say many men don’t have their dignity is that they can’t even punish their children. When you have a young girl, you can’t ask why she arrives at home late in the night. The girl can’t respect her father who is not able to give anything as she gets everything she needs from her mother as the one receiving the money from UNHCR. The men don’t have control over the behavior of their children and they can’t punish them. In the past, if there was a misunderstanding in the family, the final decision was made by a husband, but now the situation has changed and the final decision is made by the wife. So, without going into details, the majority of men feel they have no dignity*. Male respondent, FGD with community members, Mukarange.

The above statements were confirmed by female respondents who highlighted that for men to have dignity, they need to be able to provide for their families by finding a job and/or owning a business instead of spending the day sitting at home in the refugee camp.

*According to my observation, men who have their dignity are those who have a job or business so that they bring income to the family. Those ones can manage their households well. But for men who stay at home or sit at the compounds waiting for meals, they are despised and don’t have dignity in their families*. Female respondent, FGD with local and religious leaders, Mukarange.

The situation in the camps often prompted low self-esteem among men who, if not supported and/or not able to adopt positive coping mechanisms, were drawn into hopelessness. In a few cases, this led men to commit suicide to end social suffering, drinking alcohol and/or taking drugs to overcome sleeplessness or separating from their wives and engaging in other unions. Conflict between spouses sometimes impacted the children as well. For instance, in one family, a man left the house and went to live with another woman, thus leaving behind his wife and 10 children in the camp. The husband positioned their daughter (still studying) as the head of the household instead of his former wife. That daughter became the leader of the family who was entitled to get the family monthly allowances. The mother was offended and felt she had no value, which prompted her desire to commit suicide since she believed that she was supposed to take care of her children instead of her daughter.

For many male respondents, the issue of gender equality and changes related to gender roles and responsibilities were the main factors that could lead to suicidal ideations among men. Indeed, this was reported to a much higher extent as a cause compared to their past traumatic experiences in the Congo and current poor life conditions in the Rwandan refugee camps.

*It is not only poverty that causes suicide, but conflicts that exist in families. We can, for instance, go cultivating and when we come back, I can pass somewhere on the way and she can go home quickly. When I am back home, I find her drunk. When I ask her why she did not cook, I am insulted in front of the children. That will make me sad and I can kill myself*. Male respondent, FGD with refugee committee of elders and vulnerable people.

*I joined sociotherapy because I was experiencing bad moments when I was having intense conflicts with my wife. I sought help, starting from the village level, and subsequently from the president of the camp, Plan International and MINEMA^4^ office. Even when I participated in the training of Abunzi* [mediators], *I shared my case in order to seek advice. At that time, I had spent three months living alone for the sake of my own safety. My wife despised me, saying that I did not build the house we were living in and I did not bring anything to the family. Even though I have bought four fields to cultivate, but she did not give me any respect. I felt like a person without any respect in society. I thought about committing suicide because you imagine yourself as a man who spends three months alone in a house, preparing himself the meals, is not easy. I spent all night without sleeping, thinking about our marriage, how I loved her and how now she abuses me at an old age. All this made me feel psychologically traumatized*. Male respondent with suicidal ideation, Nyarugenge.

While not many respondents highlighted the issue of sex deprivation in their relationship as a potential cause of suicidal ideation and behavior, one male respondent clearly mentioned that he felt terribly insecure and no longer a man when his wife decided to separate their beds and accused him of being a drunkard. According to this male respondent, his wife’s reason for this deprivation was an excuse since he had also drunk alcohol when they were back to Congo. He attributed his wife’s behaviors to the fact that he is no longer able to financially provide for his family.

*What caused that* [suicide attempt] *is that since we reached this camp, she denied me my rights as a husband. She separated beds, she had her own bed, and I had mine. After bearing with that for 4 years, I wondered whether I am still a real man! She said that I drink and that she doesn’t want me to use alcohol. However, when we got married in Congo I already used to drink. I don’t think drinking was the cause. Instead, it was poverty. I tried suicide this month (*July*) because she provoked me with bad words in addition to what I was going through, and I got very annoyed. She told me “remember a man is respected by the property he owns!” When I heard this, I got very annoyed*. Male respondent with suicide ideations, Nyarugenge.

A staff member of an NGO working in the camps also linked the issue of gender equality with sex deprivation:

*What is more important is spousal differences in understanding gender equality, because we have many cases of men who committed suicide caused by a misconception of gender equality. If a woman starts a dispute, her husband cannot beat her fearing to be arrested; that is why men prefer to die. In some cases of family conflicts, couples sleep in separate beds*. Male respondent, FGD with stakeholders, Nyarugenge.

#### Management of Financial Resources (mVisa)

The loss of manhood was exacerbated by the recent change in the way support for basic needs was provided in the camps. From September 2017 onwards, the Rwandan branch of the UNHCR and the WFP introduced a new cash payment system (locally known as mVisa) that has been generally well received by the refugee community.^5^ Almost all interviewees testified that the monthly allowance of 7,500 Rwandan Francs (USD 8.50) per individual is often used to cover their basic needs such as buying food, cooking materials, clothes, or renting a piece of land for cultivation. However, almost all respondents reported that the allowance is not sufficient to cover their needs and so they try to cope obtaining small community loans refunded on a monthly basis. In general, women are in charge of the family’s financial management and monthly allowance since when families initially registered to receive UNHCR/WFP food, the women were generally designated as “head of household”. The change from a food to cash support system was not wholeheartedly accepted by men. Some men described having their position of family providers replaced by UNHCR/WFP⟶a situation that humiliated them in front of their wives or families.

*No, we don’t have our dignity as men, as other men. I experienced this after arriving in this camp. A wife is not yours and, a daughter is not yours, and children are followed by specific organizations. You can’t have a time to discuss with your wife and decide together what to do because she values mVisa and UNHCR*. Male respondent, FGD with community members, Mukarange.

*Aaah, as I see, men living in this camp don’t have the right to that money. You see, if someone has the habit to take a beer, he cannot use that money. Previously, the last time when WFP distributed food, oil, etc., a man could take a bottle of oil, sell it and have money to buy a beer. But now, WFP gives money to the head of the family and in most cases heads of families are women. So, if one tries to take some money in order to buy other things, this can create conflicts in the family. So, for men the situation has changed and they have to be aware of that and try to manage that situation*. Male respondent with suicide ideations, Nyarugenge.

According to some respondents (men and women), it was not a good option to have men be responsible for WFP money, since some men used the money irresponsibly (i.e. buying alcohol) and not meeting the family’s needs, which created tension at the family level. On the other hand, women managing the WFP allowances were not welcomed by men who considered the women to be selfish because they would not share a bit of money to cover their (the men’s) needs and/or allow them to buy a single bottle of alcohol. Coupled with the existing challenges in family communication and changes in gender dynamics, financial issues often led to a high level of family conflict and in some cases incited thoughts of suicide among men.

*We didn’t receive any case of suicide last year* [before December 2017], *because they received food instead of money. Back then, a man could not ask about food, he could eat, but did not go in depth asking details to his wife. In their culture, the man receives money in his hands, but today money is received by his wife and it is difficult for a woman to give her husband the money to buy a beer. I think that ideas of suicide and family conflicts increased since last September* [2017]. A refugee local leader, Nyarugenge.

*As mentioned before, a man spends his day at a place named “ku gahinda”, which means sorrow mountain, and when he arrives home he doesn’t take time to talk with his family* [wife and children]. *The wife on her side also doesn’t converse with their children and doesn’t plan with her husband on how to use the ration* [money] *once they have it. When the man asks money from his wife, as she is the one to manage it, the wife doesn’t give it because it is reserved to feed the family. Hence, the man feels neglected and conflicts start that lead even to suicide. Often, men commit suicide when the monthly allowance is obtained. Men don’t feel any taste for life, they consider themselves as useless*. Female respondent, FGD with community members, Nyarugenge.

Some female respondents disagreed with the male accusation of disobedience, since for them, the little money received from WFP barely covered their family’s needs.

*You see, our husbands accuse us of disobeying them, but it’s not true. It is like this, I might give my husband the money to go and do the shopping, but he might not buy what he is supposed to buy. Therefore, I will have to do it myself. So, if I bring the food home, he thinks that I have disobeyed him. This is unjust. We are given this money by WFP for food only, so if you happen to use it for other things like buying sandals, you don’t understand what WFP provides the money for. So, if he is an irresponsible man, you end up quarrelling for nothing, thinking that you disobey him while you are just confused of what to do. The quarrel is brought in due to insufficient funds*. Female respondent, FGD with community members, Nyarugenge.

There were, however, some respondents who reported that marital partners do sit and decide together on how to manage the income they receive. In these families, the women who receive the monthly allowances are aware of their husbands’ needs such as buying a drink with friends, which is culturally very meaningful since the men are then able to socialize with peers while sharing drinks.

*To respect my husband requires that he lets me manage the money as I managed the food we received before. However, I have to be aware that my husband needs a little money to satisfy some of his needs such as sitting with friends and taking a bottle of alcohol*. Female respondent, FGD with community mobilizers, Nyarugenge.

The respondents noted that gaining employment or initiating income-generating activities was difficult while living in the refugee camps. However, a few respondents did manage to secure paid jobs in organizations working in the camps such as Plan International, ARC, World Vision, CBS/DIR, and AHA. A few refugees managed to open small businesses such as selling food crops and running small restaurants, which partly contributed to meeting their family’s daily needs.

#### Inability to Fulfill Parental (Motherhood) Responsibilities

A few old women who spoke about suicide ideation or attempted suicide reported their fear of not being able to cover their grown children’s needs and the consequences that might follow from that situation (their adolescent and young adult children engaging in sexual relationships to cover their needs). In a position wherein they could not fulfil their responsibilities as a parent, these elderly mothers observed their grown children (especially daughters) misbehaving, which incited them to envision suicide instead of continuing their (the mothers’) suffering.

*I have six girls. One of them asks me lotion, another one asks me shoes, again another asks me clothes like other girls of their age, while I am not able to provide this. That is why you see most girls here getting pregnant at such a young age. When a girl meets with a boy who gives her 1,000 Frw to buy lotion, she easily accepts his propositions including having sex. Imagine you are a parent and your daughter comes with all those things and later becomes pregnant just because of poverty, won’t you feel pain in your heart? It hurts you as a parent to the extent you feel like committing suicide rather than living such a life*. Female respondent with suicide ideations, Mukarange.

A woman who attempted suicide narrated:

*That day* [of attempting suicide]*, we* [she and her daughter] *had a lot of misunderstandings and I was angry. I said that she could meet her boyfriend outside my home, but not inside. That was my reason of being angry. You know, she brought him during the night and they wanted to sleep together while I slept in my room; that was very bad to me. I could not tolerate that situation. Can you tolerate that a boyfriend comes and sleeps with your daughter while you are in the same house? And that man is not even her legal husband. No. That is very bad. My daughter has a child and now she is pregnant again from the same boyfriend. Better they live in their own house, and then we visit each other*. Female respondent, Nyarugenge.

In some cases, the fear of not being able to cover the family needs due to their limited monthly funds outweighed the desire to live as also expressed by a young girl whose coping mechanisms were limited:

*You see, now I am a girl and stay at home without any job. I am an orphan living with my young sisters. I felt that I could not be the head of the family, not feeling able to take that responsibility. I was thinking that it could be better to commit suicide and let my young sisters suffer themselves without me. I was not able to cover all that we needed, I was feeling without safety, and so on*. Female respondent with suicide ideations, Nyarugenge.

#### Unmarried Pregnant Young Women With Suicide Ideation

Becoming pregnant without being married is generally not culturally accepted by Congolese families and community members living in the Rwandan refugee camps. This situation often leads to discrimination and negative labelling (i.e. prostitutes), which is why girls with unwanted/unplanned pregnancies frequently become isolated with limited contact with the rest of their family and community members. These girls may express thoughts of suicide as evidenced by two in Mukarange camp who narrated that they found themselves emotionally devastated with no hope for the future. They dropped out of school from self and social stigma and, more importantly, struggled with wondering how they would take care of their children while they (the girls) were also still young. They also reported lacking family support and facing family and community insults after people learned of their pregnancies.

*I got pregnant while still young, I was a student. I did not plan that. Then I saw my living conditions here in the camp and my family not having means to support me. I was thinking that if I told them my problem, they would not understand. I was thinking that it could be better to commit suicide instead of causing problems to my family. I stopped going to school. When you have such problems, you prefer to be isolated, you feel hopeless. I spent much time thinking of how I could take care of a child being still young. When people started to see that I was pregnant, they observe that you have changed, but do not tell you what they observed. I could not sleep; I was very concerned that my family is worried because of me. I could not meet or talk to people like before, I became someone living in isolation. I felt stressed as my family wanted to know the father of the baby. It was a bad situation and I was challenged to give them the answer. I was thinking that if I tell them who impregnated me, that boy will be taken to prison while he did not take me by force. After they* [parents] *calmed down, I told them who the father is*. Female respondent with suicide ideations, Mukarange.

#### Use of Alcohol and Drugs

The use of drugs and alcohol was not commonly reported by respondents with suicidal ideation. Some said that they had stopped drinking alcohol when confronted with life challenges. Only a few respondents conveyed that they sometimes drank alcohol to cope with their sleeping disorders.

*No, that did not happen to me; on the contrary, I stopped taking alcohol because I was worried about the life of my children. It is difficult to live in these conditions and take alcohol or drugs*. Male respondent with suicidal ideations, Nyarugenge.

*At that time* [when his wife was sick and hospitalized]*, I could meet my friends and they asked me the evolution of my wife’s illness, and then they said: “We can offer you a bottle of beer,” and when I took that, I could get sleep for a short time during the night*. Male respondent with suicidal ideations, Mukarange.

However, the family members of these respondents reported that there was alcohol and drug abuse among people with suicidal ideations and behavior, which contradicted the respondents’ statements noted above.

### Signs, Symptoms and Perceived Behaviors Prior to Suicide

According to some respondents, it is often difficult to know that a person will commit suicide despite the changes observed in the person’s attitudes and behaviors. They reported that some victims tended to “act very normal” prior to or during the day of suicide, probably as a way of not attracting the attention of family members. For instance, victims who committed suicide were found to be happy, relating well with family members, attending church services or playing football on the same day that they committed the act.

Symptoms commonly observed referred to mental disorders, such as depression (*agahinda gakabije*). The presence of these symptoms often went unnoticed by refugees, which resulted in the respondents in this study pointing to the need for services to focus on early detection of depression. Symptoms observed by the respondents included anxiety, isolation, a lack of interest in communal activities such as church attendance, loss of hope for a future life, silence about one’s feelings, crying (mainly among women), insecurity, alcohol abuse, deep sadness, hatred towards people of the opposite sex, a loss of dignity and value (mainly among men) and a range of psychosomatic symptoms including stomachache, chronic headache, heart palpitations and insomnia. Loss of interest in one’s own physical appearance and in doing daily activities was also commonly mentioned.

### Community Perceptions of Suicidal Ideation and Behavior

Respondents expressed having mixed feelings about committing suicide in refugee camps. Suicide is known in the camps as “*gutora supanet*”, which literally means using the rope of a bednet [as a lethal means for suicide] and is the most commonly used language for this action. For some, suicide is abominable, and all the more so since it has negative consequences for the entire family because of stigma and conflict. However, for others, committing suicide was saluted and considered to be an act of courage, which some of them in a similar situation have failed to adopt as a response to their severe emotional pain.

*Sometimes when a man dies, people say that he is a hero because he chose to commit suicide instead of being mistreated by his wife*. Male respondent, FGD with community mobilizers, Nyarugenge.

Consequences resulting from suicide ideation and behavior range from emotional suffering, family disruption and social stigma to increased economic problems for those who have had suicidal ideations and attempts. Family members of those with suicidal ideation often felt unhappy and unsafe, fearing that a suicide attempt might happen any time. Feelings of shame and guilt among those who had attempted to commit suicide and their families were often shared. Members of these persons’ nuclear families face stigmatization by extended family members and/or the community since they are perceived as the trigger that led to their relatives’ suicidal ideation and behavior. In single-parented families, a major consequence of adult suicide is the issue of the children’s care following a parent’s suicide. This often results in the children being placed across several families throughout the camp.

*Yes, for consequences, let me start with my young sisters. When they observed that I had anger, they were unhappy, worried and felt insecure because of me*. Female respondent with suicide ideations, Nyarugenge.

In some cases, families have also been affected economically by a relative’s suicide since those who committed suicide were part of a larger family support system.

### Support Mechanisms to Address Psychosocial Issues Related to Suicide

Respondents highlighted that whenever family conflicts and/or psychological issues are noticed within a family, the first support usually comes from family members and neighbors. This support is organized through family meetings during which various matters, such as family conflicts, are discussed to find solutions. Individuals facing psychological issues are comforted in these meetings. Community meetings such as parents’ evening programs (*umugoroba w’ababyeyi*) were mentioned by a number of respondents as another platform for discussing individuals or families’ psychosocial issues. However, they stated that this is not done on a regular basis. Most of the respondents referred to the sociotherapy program in the camps for sharing and healing in a group setting and individual counselling services provided by the Rwandese Association of Trauma Counsellors (ARCT) for individuals with psychological distress who can also be transferred to the camp health centre or district hospital if needed.

*I was feeling hopeless and I was thinking on suicide. I was very tired with problems* [lack of means] *and wanted to commit suicide. But, after being in sociotherapy, I changed my thinking. As I have been helped by sociotherapy, I am also able to help other people with psychological problems*. Female respondent with suicide ideations, Nyarugenge.

Community volunteers supervised by the American Refugee Committee (ARC) staff were also mentioned as key people in the community for family conflict resolution initiatives such as mediation. Whenever conflicts were deemed to be severe and unresolvable, respondents highlighted that camp managers opted for temporary separation of the family members, for instance, by providing an additional house.

Faith-based support was also commonly mentioned by respondents. This support generally involved family visits when something bad happened. The visits included an exchange about the word of God, praying together and the provision of supplies for basic needs, namely food. Most of the refugees in both camps are members of the Seven Day Adventist Church, hence they benefit from Church support. However, some respondents reported that *Eglise des Amis* (Friends of Peace House Program)** ^6^ ** provides training to refugees and the host community (Rwandan population living close to the camps) on conflict resolution as well.

All participants appreciated camp-organized activities and events for physical recreation and emotional relief mainly targeting children and youth: games, concerts, dances, sketches, watching football, or movies. For old men, cultural games such as chess and stick fighting (*Kuyobanwa)* and, for women, marathons (*Gukataza*) are regularly organized and help to release their stress.

While respondents seem to be aware of the various community mechanisms for support of individuals and/or families with psychosocial problems (e.g., community volunteers), it was not always clear to the refugees how to proceed when additional support was needed. This was partly due to the fact that some respondents were not aware of the multiple organizations working in camps and their areas of intervention.

*In general, people are aware of the referral system for managing social problems; a system that runs from the village to the executive committee of the camp and to stakeholders in charge of managing problems in the camp. But, the referral system for caring for people with psychological problems is not clear for many people. I am not aware of that referral system. I am among the people who were traumatized psychologically, but I did not receive that support nor a referral to another level*. Male respondent with suicide ideations, Nyarugenge.

### Recommendations

Although support mechanisms are available, respondents provided recommendations to overcome life adversities leading to suicide ideation and behavior in both camps as highlighted below.

#### Community Awareness of Suicide and Prevention

Stakeholders working in camps should collaboratively plan and conduct regular community campaigns that raise awareness of the potential triggers for suicide. The campaigns should focus on recognition of preliminary symptoms of suicidal ideation; individual, family and community consequences of suicide; and the promotion of support- and care-seeking among refugees. It would also be beneficial to organize training for camp community members on the basic support skills for people who experienced suicidal ideations or have attempted to commit suicide.

*It will be better to sensitize people to share their problems with others and not keep them for themselves. When you share your problem with someone, you can get advice and change your dangerous thoughts. Community members should also be sensitized to identify people with psychological problems and report them to sociotherapists or to other levels in the referral system to be cared for. For instance, if you identify a person with psychological problems, you can help him/her and if you are not able to do so, you can refer him/her to other levels of the care system where he/she can get appropriate care*. Female respondent with suicide ideations, Mukarange.

#### Psychosocial Support

The fact that people who experience suicidal ideations and those who have attempted suicide, and their families, were found to be highly affected by suicide at an emotional, psychological and socio-economic level, requires that specific support be tailored to individuals, families and the community as a whole. Based on the study findings presented here, this can be done through:

- The availability of individual and family counseling services in cases when suicidal ideations and/or attempts have been reported. Counseling sessions to help victims (both victims of ideation and behavior as well as their family members) release their pain (emotional suffering) and overcome fears, and to promote positive ideas about their future life while preventing potential suicide contagion within families.
- Increase psychologists and psychiatric nurses in the personnel of the clinic in the camps.
- Reinforce trainings for the health personnel with a particular focus on mental health (i.e. depression, drug and alcohol abuse, suicidality, etc.).
- Create special provisions for people with mental disorders to access care at the referral hospital.
- Strengthen community-based services such as parents’ evening programs (*Umugoroba w’ababyeyi*) and ensure they are being held on a regular basis. These programs promote community sharing about their everyday lives and build community trust that can help refugees overcome life challenges (both individually and as families).
- The sociotherapy program implemented in the camps was highly recommended by respondents for healing, promoting positive family and social relationships and improving the coping mechanisms of individuals and families. Scale-up of the program through an increased number of trained sociotherapists and facilitated sociotherapy groups would produce a wider societal impact including a reduction of suicide ideation among participants.
- A special focus in any activity must be to involve men, since they are much affected by current life in the refugee setting. Men were found to be traditionally characterized as strong human beings who could not show their emotional suffering or ask for help if needed. To overcome this barrier, tailoring men’s involvement by developing self-help materials and forming homogenous group dialogues (only men), should be considered to facilitate an open environment for honest and non-judgmental sharing.

*It will be better to increase the number of community members in charge of taking care of people with psychological problems. I want to thank Mvura nkuvure* [Kinyarwanda term for sociotherapy— “you heal me, I heal you”] *for operating here. I really appreciated it so much. I wish that they let us continue going there, because if we put it aside, there will be setbacks. Normally, when we are down there talking to each other, you feel happy. When the time was approaching to meet, we all used to work at home very fast and wash clothes faster, so that we would not be late. When I heard that it’s over, I started wondering how things are going to look like. Now, I don’t know who can listen to me because I fear sharing with my neighbors who can gossip about my problems. In sociotherapy, we found a safe place where they normally teach good habits. I would like to ask people in charge of the sociotherapy program to take some measures, which can help those who graduated from the program to avoid setbacks. Take the example of a hoe, the one that digs is always sharp but the one that does not, gets rust*. Male respondent who previously reported suicidal ideation, Mukarange.

#### Improvement of Family Communication and Understanding of Gender Equality

Most family conflicts that led to suicidal ideation among men, in particular, were related to a lack of family communication and misconceptions of Rwandan gender equality policies and practices. In collaboration with stakeholders, it was recommended that organizations working in both camps design a culturally sensitive program that addresses the issue of gender-based violence in refugee settings. The program should include the promotion of women’s rights and empowerment while preventing men’s feelings of a loss of dignity from non-participation in family income management.

*If a couple takes their time to plan the use of money given by WFP, the spouses should not have a conflict because of money. I suggest to discuss the use of money/ration because the problem is not really the mVisa as such*. Family member of a person who committed lethal suicide, Nyarugenge.

*The lack of dialogue on the use of a mVisa card is the root cause of problems. Women feel that they have the same rights as men. There is a misunderstanding of what gender balance is. Women don’t tell their husbands how they spend the money they receive. In some families, men are not aware of the way wives spend the money, they don’t have all details. Men presume that women are the chiefs of families as they are the ones to decide. The lack of dialogue is really the cause of conflicts and should be dealt with*. Male respondent, FGD with community mobilizers, Nyarugenge.

#### Joint Stakeholders’ Mechanisms and Effective Program Delivery

Stakeholders, including NGOs, MINEMA, UNHCR, and WFP, in both camps focus on distinct areas of intervention. Thus, it was recommended that joint meetings and interventions are needed to ensure prompt actions and better outcomes. Regular sharing of information among NGOs, multilateral organizations, and MINEMA and establishing a proper referral system and follow up of referred cases were mentioned as important elements to ensure that camp communities receive effective services in an efficient manner.

One of the concerns expressed by respondents was the adoption of non-culturally relevant solutions to issues identified in the camps such as family conflicts (i.e. offering a job to one of a couple in conflict). According to the respondents, this aggravates conflict rather than ending it. More consultation and participation by community members in conflict resolution initiatives are needed to ensure that the cultural context in which the programs are embedded and implemented is considered. This would reduce community prejudice sometimes attributed to camp stakeholders.

*If conflicts are solved within a family by organizations in charge, women should not be given a reward because of accusing husbands. If I give an example, a woman might get a gift of Kitenge* [an African fabric for women] *or offered a job because she dared to accuse her husband or because of the way she responded to family conflicts. This is not appreciated by the community here in the camp*. Female respondent, FGD with community mobilizers, Nyarugenge.

*Another thing is how these organizations deal with our problems. I can submit my problem and it takes three to four months to get a solution offered. I am wondering if there is no way to solve our problems quickly. If you receive a problem and you are not able to handle it, you can refer it to one who is capable of solving it. If you don’t do it in time, you* [organizations working in camp] *will take the initiative to do it while it is not needed; that is why I beg you to work as a team. That will help us to get the solution and to prevent the scandal that can happen*. A church leader, FGD with stakeholders, Nyarugenge.

#### Economic Empowerment Through Small Income-Generating Activities

Most of family conflicts were related to insufficient financial funds provided to families in refugee camps. While obtaining access to additional funds was challenging, some families and/or individuals managed to find small paid jobs or engage in agricultural activities. However, these refugees recognized the importance of regrouping themselves into cooperatives, which they said are effectively working in their host community to ensure access to financial funds and reduce conflict. Thus, it is suggested that MINEMA in collaboration with other stakeholders, namely UNHCR and the WFP, conducts a financial analysis adapted to the refugee setting if increasing monthly allowances is challenging. Stakeholders working in camps need to leverage existing human resources among the refugee population for all job openings before considering external [non-refugee] candidates.

#### Active Involvement of Church-Based Initiatives

Many respondents recalled the important role the church has been playing, from care for spiritual wellbeing to social support. Churches organize regular activities such as prayers and events (*ibitaramo*), but churches were also found to be important sources of spiritual, emotional, social and economic support for members in need. Therefore, it was recommended that churches should keep advocating for behaviors that are considered to be physically and mentally beneficial to the community.

#### Education on Sexual and Reproductive Health Among Youth

Some of the young girls manifested suicidal ideations after becoming pregnant at a young age. For them, early pregnancy leads to a lack of family support and community stigma exacerbated by the general poor financial situation observed among the refugee population. The incidence of unwanted pregnancies among this young generation of reproductive age suggests that they are engaged in unions that often lead to sexual activities, while their knowledge of reproductive health (e.g., family planning), is still limited. In collaboration with stakeholders, a special education program on reproductive health among adolescents should be designed and implemented.

#### Promotion of Entertainment Activities

Respondents in both refugee camps showed appreciation of the activities and events organized for recreation and to reduce social isolation. Therefore, there is a need for scale up of these interventions. Most highlighted activities, particularly for the youth, included games (e.g., football tournaments), concerts and traditional dances. Activities for men included playing a traditional board game and the aforementioned “*Kuyobana*” that both symbolize a cultural way of fighting in an atmosphere of joy.

## Discussion

The study findings presented here provide a synopsis of proximal factors that may contribute to suicidal ideations, attempts and actions in both Rwandan refugee camps for Congolese refugees in general. Most of factors identified are similar to what has been reported elsewhere as listed in the Introduction, but also, as stressed by respondents, culturally specific ones. The exposure to norms and values in the host country they are unfamiliar with increases suicide risk.

Suicide (ideations, attempts, and/or death) among the Congolese refugee young generation were mainly explained by a lack of hope for the future and unwanted motherhood, in addition to their current poverty in the camps. Suicidal ideations for women were explained by an inability to cover their family’s basic needs and sometimes the misconduct of their adolescent and young adult children (i.e. drug and alcohol abuse among boys and culturally unacceptable and unwanted pregnancies outside of marriage for girls). For men, the main issue highlighted was the cultural changes they were exposed to in the camps and their implications for gender roles and responsibilities. The deprivation of their role as family provider and the empowerment of women resulted in feeling a loss of value and dignity that often led to family conflict, depression symptoms and eventually suicidal ideation. While community support mechanisms to tackle the community issues were identified, some support provided by camp organizations were found to be culturally insensitive (e.g., rewarding one of a couple in conflict and not the other), which may serve to create additional challenges refugees have to struggle with instead of solving family conflict.

Klonsky and May (13) argue that traditional theories to understand suicide have failed to differentiate between explanations of suicidal thoughts and of suicidal behavior. According to the authors, a critical advance occurred when Joiner (18) introduced the “ideation-to-action” framework, which made that differentiation. Klonsky and May (13) position their own theory, the Three-Step-Theory (3ST), within this framework, and by doing so further support the work of those who had initially created the ideation-to-action framework, e.g., Van Orden et al. (12). In short, the 3ST hypothesizes that a combination of pain (usually psychological pain) and hopelessness is required to develop and sustain suicide ideation. Step two of the 3ST suggests that ideation escalates when the pain exceeds or overwhelms connectedness to loved ones, valued roles, or any sense of meaning or purpose. If the pain exceeds connectedness, suicide ideation increases from modest/passive (e.g., “Sometimes I wonder if I would be better off dead”) to strong/active (e.g., “I would kill myself if I could”). Step three suggests that strong suicide ideation progresses to action when one has the capability to make a suicide attempt (whether lethal or non-lethal). Klonsky and May (13) stress that all factors that traditionally have been identified as risk factors for suicide remain relevant, but in a specific way, through their effects on pain, hopelessness, and/or connectedness.

Our qualitative study results give ample indication that the 3ST would be a useful framework for understanding suicide in a refugee setting. In addition, it could guide follow-up research, on specific factors (like those presented here) for explaining the trajectory from suicidal ideation to action, and contribute to effective prevention and intervention strategies in refugee camps in Rwanda. We suggest that follow-up research should take a mixed method approach with a larger study sample than presented here.

The life stories of refugees collected by sociotherapy program staff working in the Rwandan camps, our results presented here, and the pre- and post-intervention surveys of participants in sociotherapy groups (graduates) support the relevance of the 3ST to explore the development of suicidal ideations in refugee camps and progression to suicidal action. Sociotherapy staff and sociotherapists in the two camps explored in this study were asked to write the life history of a sociotherapy graduate who they considered to have a most significant change due to their participation in a sociotherapy group. The exercise resulted in 20 stories. In three of the seven stories selected by a committee for publication, suicide was featured (19). These stories are presented here in summary:

Rose, (54 years old), whose husband and three children were killed in 1995 in the war in Congo presented with a history of multiple displacements and had reached the point that she decided to commit suicide by taking rat poison. Her only son, born in 2001, noticed that something was wrong and informed his mother’s best friend. That friend convinced Rose to not go forward with her plan for the sake of her son’s future. However, Rose’s thoughts about committing suicide remained. Later, participation in sociotherapy diminished Rose’s pain, gave her hope for the future, and connected her to people with whom she could share her pain and daily life challenges.

Mbabazi, (31 years old), also had a traumatic history. She had tried to torture herself to death by starvation over two months, but to no avail. Once in the camp she isolated herself completely. One day, discovering she was pregnant by a man she was not married to, she got up early in the morning and went to the river with the intention to throw herself in. However, the thought of her children being left without someone to care for them made her struggle with her decision and finally return home. Mbabazi’s participation in sociotherapy empowered her desire to care for her children again, removed her self-blame and allowed her to socialize with others again.

Pierre, (35 years old), attempted to commit suicide while hospitalized for serious wounds incurred during the war in Congo by taking an overdose of medicines he had been prescribed. Due to his mother’s interference, he was given an antidote and he survived against his will. While in the camp, life meant nothing for Pierre. His wife had left him and took his children with her. He was without hope and afraid of other people. He detested himself and had no self-confidence. Sociotherapy eventually gave him peace of mind again and friends with whom to socialize.

All three stories confirm the hypothesis of the 3ST that pain, hopelessness and a loss of connectedness combined with the capability to commit suicide (having a bednet, rat poison, medicine at hand or a river nearby) are a potentially lethal combination. Both Rose and Mbabazi had strong suicidal ideation, but, their connectedness with others made them decide not to attempt suicide. Pierre, feeling completely isolated, did make the step to attempt suicide, since he had the capability to do so. All three stories also confirm that if one has hope that the pain can be diminished with time or effort and connectedness with others has been restored, one’s focus will be on achieving a better future rather than suicide. By participating in sociotherapy, where they first started to experience connection with others and being taken care of by these others, the three protagonists in the stories experienced a mitigation of their pain and restoration of hope for the future again. Positive changes from participating in sociotherapy were reported during the quantitative study mentioned earlier in this article. These changes were mainly related to the respondents’ psychological health, grading of their quality of life, hope for the future, coping mechanisms, and perceived social support. These outcomes justify the relevance of psychosocial programs in general in Rwandan refugee camps. The quantitative study conducted among sociotherapy participants, which found that 9% of the 98 participants enrolled in the pre-intervention survey reported suicidal ideations, supports this justification. On the one hand, this relatively high percentage can be explained by considering sociotherapy participants as a kind of clinical population with more problems than most camp refugees. On the other hand, the camp population receiving sociotherapy is relatively small (about 300 participants). It is likely that given the need expressed by refugees and stakeholders for more sociotherapy in the camps that many more refugees share the same problems as the sociotherapy participants. This likelihood is confirmed by Bell et al. (20) who found that approximately 8% of a sample of 810 women living in a camp for Congolese refugees in Rwanda (names of the camps not mentioned), who participated in a study in which a brief screening tool (SRQ-5) for women’s health assessment was tested, reported suicidal thoughts in the four weeks prior to taking the survey.

Suicides are preventable if a range of measures are taken at a population, sub-population and individual level as indicated in stories above. For effective suicide prevention, one needs to know the risk factors that contribute to thoughts of suicide (suicidal ideation), attempted suicide and actual suicide (1). In the same framework, a findings dissemination meeting (with community members, camp managers and stakeholders) organized at the end of our study recommended to design an implementation plan including regular sensitization campaigns against suicide and targeting the locally and culturally known “mountains of sorrow” that camp inhabitants experience with interventions that promote the psychosocial wellbeing of all refugees, especially men. In addition, the stakeholders proposed that gender equality across both camps should be carefully explained while being promoted to prevent misunderstandings and/or misconceptions as well as revisiting the system of rewarding those who report gender-based violence to ensure that the system is not negatively perceived by some refugee community members. Moreover, consideration of the community’s feedback on program designs, exploration of the legal aspects of suicide in Rwanda, improvement of the referral system for suicide cases and the expansion of the psychosocial programs already in place in the camps (i.e., sociotherapy) were strongly recommended. Since the problem of suicidal ideation and attempts came to light between January and June 2018, organizational collaboration and sensitization meetings were conducted to raise awareness of the impact of suicide. Community members discussed suicide, made decisions, and explored preventive strategies. They also discussed their experiences with the negative impact of suicide on families. As of November 2018, when this study’s findings were disseminated, no further suicide attempts or deaths have been observed in either Rwandan refugee camp.

## Conclusion

Our research showed that a haunting painful past that severely affects the psychosocial wellbeing of refugees, coupled with daily life challenges (family conflicts, misunderstanding of gender equality, unwanted pregnancies, poverty, etc.), increases lack of hope for the future and a sense of isolation, and is a risk factor for developing suicidal ideations, attempts and actions. Community support mechanisms available to address issues identified by respondents were highlighted. However, some of the support systems in the camps were found not always culturally sensitive, hence the need for adaptation to the local context. While the research has provided some recommendations on how to mitigate the incidences of suicidal attempts and actions, an additional quantitative study is suggested to determine the magnitude of suicidal ideations in both refugee camps.

## Data Availability Statement

Due to ethical considerations, datasets cannot be shared with third parties. Requests to access the datasets should be directed to the corresponding author.

## Author Contributions

CI participated in the conception and design of research, field activities and data analysis and wrote the research report. CI and AR converted the research report into an article. Both authors have approved the final manuscript.

## Funding

The Deutsche Gesellschaft für Internationale Zusammenarbeit (GIZ) (83284449/02/2018) provided the financial support for the implementation of community-based sociotherapy in the camps that served as the context for this study.

## Conflict of Interest

The authors declare that the research was conducted in the absence of any commercial or financial relationships that could be construed as a potential conflict of interest. 

## Footnotes

1 Pseudonyms have been used for camp names for ethical purposes.

2 Community-based sociotherapy aims to contribute to psychosocial wellbeing, reconciliation and social cohesion in the aftermath of mass violence. It implements a socio-dynamic approach, using the group as a therapeutic medium in the establishment of trust, the creation of an open environment for discussion and the formation of peer-support structures. People meet in their neighbourhood for weekly sessions over a period of 15 weeks facilitated by two trained sociotherapists who are from the same neighbourhood [see e.g. (14, 15)].

3 The 74 refugees in the post intervention are less than the number of refugees enrolled in the pre-intervention (n = 98) because only sociotherapy participants who attended at least 10 of the 15 sociotherapy sessions were eligible for post-intervention evaluation. Five were not eligible for the post-intervention (attended less than 10 sociotherapy sessions), and 19 could not be located (i.e. left the camp, gone back to school, etc.) during the time of the post-intervention survey.

4 MINEMA: Ministry of Emergency Management.

5 A pilot cash and voucher initiative launched by the WFP to replace the general food distribution in refugee camps. The monthly allowance (mVisa) enables refugees to pay through their mobile phones, giving families much more independence to make their own choices and diversify their diets according to their individual needs and preferences.

6 The “*Eglise des Amis*” Church’s “Friends of Peace House Program” offers services related to conflict resolution, mediation and trauma healing.

## References

[1] World Health Organization (WHO) Factsheet on suicide. World Health Organization (2018). Retrieved from http://www.who.int/news-room/fact-sheets/detail/suicide Accessed on 20 April 2019.

[2] Aoe T Shetty S Sivill T Blanton C Ellis H Geltman PL Suicidal ideation and mental health of Bhutanese refugees in the United States. J Immigr Minor Health (2016) 18(4):828–35. 10.1007/s10903-015-0325-7 PMC4905789 26711245

[3] Cohen J Safe in our hands?: A study of suicide and self-harm in asylum seekers. J Forensic Leg Med (2008) 15(4):235–44. 10.1016/j.jflm.2007.11.001:50-1 18423357

[4] Hollander AC Kirkbride,JB Pitman,A Lundberg M Lewis G Magnusson C Are refugees at increased risk of suicide compared with non-refugee migrants and the host population. Eur J Public Health (2017) 27(3)50–1. 10.1093/eurpub/ckx187.129

[5] Refugee Health Technical Assistance Center Suicide. (2011). Retrieved from https://refugeehealthta.org/physical-mental-health/mental-health/suicide/ Accessed on 20 April 2019.

[6] Voelker R. (ed.) Suicide awareness needed for Bhutanese refugees in the United States. JAMA (2013) 28(310):789. News from the Centers for Disease Control and Prevention Section. 10.1001/jama.2013.194707

[7] Wasserman D Suicide risk in refugees and asylum seekers. Eur Psychiatry (2017) 41(Supplement):S35–6. 10.1016/j.eurpsy.2017.01.167

[8] Jankovic J Bremner S Bogic M Lecic-Tosevski D Ajdukovic D Franciskovic T Trauma and suicidality in war affected communities. Eur Psychiatry (2013) 28(8):514–20. 10.1016/j.eurpsy.2012.06.001 22986125

[9] International Organization for Migration (IOM) Assessment of suicide risks and factors in a refugee camp in Thailand. (2017). Retrieved from https://progressivevoicemyanmar.org/wp-content/uploads/2017/06/IOM-Mission-in-Thailand-Assessment-of-Suicide-Risks-and-Factors-in-a-Refugee-Camp-in-Thailand.pdf Accessed on 20 April 2019.

[10] Mars B Burrows S Hjelmeland H Gunnell D Suicidal behaviour across the African continent: a review of the literature. BMC Public Health (2014) 14:606. 10.1186/1471-2458-14-606 24927746 PMC4067111

[11] United High Commissioner for Refugees (UNHCR) Rwanda Monthly Factsheet. United High Commissioner for Refugees (2018). Retrieved from https://www.unhcr.org/rw/wp-content/uploads/sites/4/2019/01/November-2018-Operational-Update.pdf Accessed on 20 April 2019.

[12] Van Orden KA Witte TK Cukrowicz KC Braithwaite ZD Selby EA Joiner TE The interpersonal theory of suicide. Psychol Rev (2010) 117(2):575–600. 10.1037/a0018697 20438238 PMC3130348

[13] Klonsky ED May AM The three-step theory (3ST): A new theory of suicide rooted in the “ideation-to-action” framework. Int J Cognit Ther (2015) 8(2):114–29. 10.1521/ijct.2015.8.2.114

[14] Richters A Rutayisire T Dekker C Care as a turning point in sociotherapy: Remaking the moral world in post-genocide Rwanda. *Medische Antropologie: Tijdschrift over Gezondheid en Cultuur* . (2010) 22(1):93–108. Retrieved from http://tma.socsci.uva.nl/22_1/richters.pdf.

[15] Ingabire MC Kagoyire G Karangwa D Ingabire N Habarugira N Jansen A Trauma informed restorative justice through community-based sociotherapy in Rwanda. Intervention: J Ment Health Psychosoc Support Conflict Affected Areas (2017) 15(3):241–53. 10.1097/WTF.0000000000000163

[16] Ministry of Gender and Family Promotion National Gender Policy. (2010). Retrieved from https://migeprof.gov.rw/fileadmin/_migrated/content_uploads/National_Gender_Policy-2.pdf.

[17] Akinyemi A (2019). ‘No sex without fighting’ - tackling toxic masculinity in DR Congo. Retrieved from https://www.bbc.com/news/world-africa-48094438.

[18] Joiner TE Why people die by suicide. Cambridge, MA: Harvard University Press (2005).

[19] Sewimfura T Ngabonziza B Mukimbili N Richters A Booklet on the Camp-based Sociotherapy. (2019).

[20] Bell SA Lori J Redman R Seng J Psychometric Validation and Comparison of the Self-Reporting Questionnaire-20 and Self-Reporting Questionnaire-Suicidal Ideation and Behavior Among Congolese Refugee Women. J Nurs Meas (2015) 23(3):393–408. 10.1891/1061-3749.23.3.393 26673766 PMC4927334

